# Association of Anxiety and Depression With All‐Cause Mortality in Individuals With Coronary Heart Disease

**DOI:** 10.1161/JAHA.112.000068

**Published:** 2013-04-24

**Authors:** Lana L. Watkins, Gary G. Koch, Andrew Sherwood, James A. Blumenthal, Jonathan R.T. Davidson, Christopher O'Connor, Michael H. Sketch

**Affiliations:** 1 Departments of Psychiatry and Behavioral Sciences, Duke University Medical Center, Durham, NC (L.L.W., A.S., J.A.B., J.R.D.); 2 Department of Biostatistics, University of North Carolina, Chapel Hill, NC (C.C., M.H.S.); 3 Department of Medicine, Duke University Medical Center, Durham, NC (G.G.K.)

**Keywords:** all‐cause mortality, anxiety, coronary heart disease, depression

## Abstract

**Background:**

Depression has been related to mortality in coronary heart disease (CHD) patients, but few studies have evaluated the role of anxiety or the role of the co‐occurrence of depression and anxiety. We examined whether anxiety is associated with increased risk of mortality after accounting for depression in individuals with established CHD.

**Methods and Results:**

The cohort was composed of 934 men and women with confirmed CHD (mean age, 62±11 years) who completed the Hospital Anxiety and Depression scale (HADS) during hospitalization for coronary angiography. Over the 3‐year follow‐up period, there were 133 deaths. Elevated scores on the HADS anxiety subscale (HADS‐A≥8) were associated with increased risk of mortality after accounting for established risk factors including age, congestive heart failure, left ventricular ejection fraction, 3‐vessel disease, and renal disease (hazard ratio [HR], 2.27; 95% CI, 1.55 to 3.33; *P*<0.001). Elevated scores on the HADS depression subscale (HADS‐D≥8) were also associated with increased risk of mortality (HR, 2.18; 95% CI, 1.47 to 3.22; *P*<0.001). When both psychosocial factors were included in the model, each maintained an association with mortality (anxiety, HR, 1.83; 95% CI, 1.18 to 2.83; *P*=0.006; depression, HR, 1.66; 95% CI, 1.06 to 2.58; *P*=0.025). Estimation of the HR for patients with both anxiety and depression versus those with neither revealed a larger HR than for patients with either factor alone (HR, 3.10; 95% CI, 1.95 to 4.94; *P*<0.001).

**Conclusions:**

Anxiety is associated with increased risk of mortality in CHD patients, particularly when comorbid with depression. Future studies should focus on the co‐occurrence of these psychosocial factors as markers of increased mortality risk.

## Introduction

Over the last 2 decades, a number of prospective epidemiological studies have provided support for an association of depression with mortality in coronary heart disease (CHD) patients.^1–3^ However, relatively little attention has focused on the role played by anxiety in depression‐related risk, despite the extensive comorbidity shared between depression and anxiety.^4–6^ Chronic anxiety is accompanied by a number of pathophysiological processes including elevated sympathetic nervous system activity,^7–9^ inflammation,^10–13^ and hypertension.^14^ Anxiety has also been associated with increased risk of cardiac mortality in prospective cohort studies of medically healthy individuals^15–19^ and in a number of CHD patient populations.^20–27^ However, not all studies have found an association between anxiety and poor clinical outcomes in CHD patients.^28–31^

The goal of the present study was to evaluate the explanatory roles of anxiety alone and of comorbid anxiety and depression for mortality risk in patients with established CHD. Anxiety and depression were assessed using the Hospital Anxiety and Depression scale (HADS), a scale developed specifically to discriminate anxiety from depression in medically ill hospitalized patients.^32^

## Methods

### Participants

**Figure 1.**
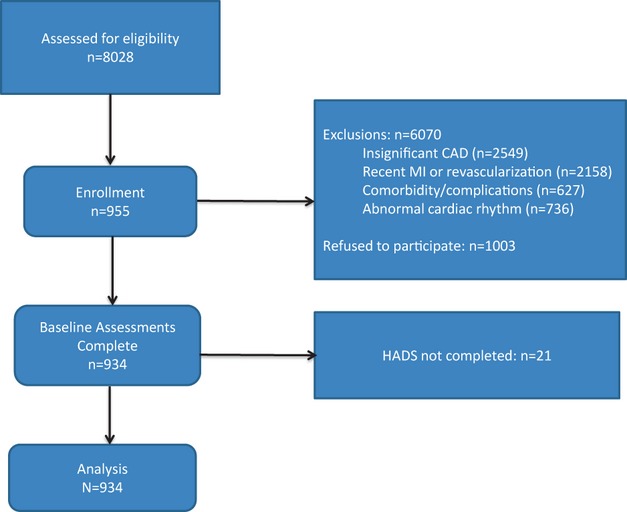
Flow chart showing numbers of patients screened, excluded, and enrolled. CAD indicates coronary artery disease; MI, myocardial infarction; HADS, Hospital Anxiety and Depression Scale.

VAGUS (Very Anxious Group Under Scrutiny) was a prospective study designed to evaluate the relationship of anxiety with risk of adverse clinical outcomes in CHD patients. All patients were enrolled at Duke University Medical Center over a 27‐month period during an inpatient or outpatient visit for diagnostic cardiac catheterization. To be eligible for the study, patients were required to have either cardiac catheterization–confirmed significant stenosis (≥75%) of >1 major coronary artery or a documented history of prior myocardial infarction (MI) or revascularization procedure. Of the 9162 patients undergoing left heart cardiac catheterization during the enrollment period, 8028 charts were available for screening, and approximately one‐third of these patients (n=2549) were excluded because of insignificant CAD. Because one aspect of the parent study focused on heart rate variability (HRV), approximately one‐third of the available patients (n=2894) were excluded because they were not in normal sinus rhythm or had a 30‐day history of myocardial infarction and/or revascularization. Eight percent (n=627) were not approached because of medical, physical, or psychiatric complications that could impact the patient's ability to communicate (eg, ventilator dependence, stroke‐related aphasia, language conflict, psychosis). Of the 955 patients enrolled in the VAGUS study, 21 patients were unable to complete the HADS, leaving a study sample of 934 (see Figure 1). Of these 21 patients, 6 died and 15 survived. The 21 patients with missing HADS data were comparable to the others in terms of all other demographics and medical history variables, suggesting that completion of the HADS is unlikely to have affected the study findings. This study was approved by the Duke University Medical Center Institutional Review Board, and all patients gave written informed consent before participating in the research protocol.

### Measures of Medical Disease Severity and Lifestyle Factors

Medical history, current diagnoses, medications, blood pressure, and coronary disease treatment plan (medical/revascularization/surgical) were extracted from patient hospital charts. A modified version of the Stanford 7‐day physical activity recall interview^33^ was used to estimate physical activity and habitual sleep duration during the 7‐day period prior to hospitalization. Left ventricular ejection fraction (LVEF) and degree of coronary artery stenosis were obtained from the cardiac catheterization report. Education status and smoking status were obtained through patient interview.

### Assessment of Anxiety and Depression

The Hospital Anxiety and Depression Scale (HADS) is a 14‐item self‐report questionnaire comprising 4‐point Likert‐scaled items covering the occurrence of symptoms of anxiety (HADS‐A) and depression (HADS‐D) over the past 2 weeks.^32^ The HADS was specifically designed to avoid false‐positives when administered in hospital settings and therefore focuses on psychological and cognitive symptoms, rather than somatic symptoms or sleep and appetite disturbance. The majority of the symptoms included in the HADS‐A are indices of generalized anxiety, with only 1 item related to panic. The depression scale primarily comprises symptoms of anhedonia. Internal consistency (Cronbach's alpha) for the HADS‐A and HADS‐D ranged from 0.67 to 0.93 in previous studies^34^ and was 0.78 for each subscale in the current sample. A 2‐factor structure of the HADS has been confirmed in a number of studies,^35–37^ and a cutoff score of 8 on the HADS‐A subscale (range, 0 to 21) and 8 on the HADS‐D subscale (range, 0 to 21) has been found to provide the optimal balance between sensitivity and specificity for identifying cases,^34^ as originally recommended by Zigmond and Snaith.^32^ Results from 1 recent study indicated that approximately 21% of community‐dwelling individuals score ≥8 on the HADS‐A subscale and 23% score ≥8 on the HADS‐D scale.^38^ The HADS has moderate test‐retest reliability (*r*>0.8), and average sensitivities and specificities of ≥0.8.^39–41^ Among the 934 patients included, 928 had no missing items, and 6 had only 1 missing item, which was replaced using the mean score of the other items on the subscale in question.

### End‐Point Ascertainment

All patients were contacted by mail at 6 months and annually thereafter up to 4 years after the initial assessment to document hospitalizations and vital status. For patients who were identified as deceased, discharge summaries, clinic notes, and supporting documentation (EKG and laboratory results) were obtained for each cardiac‐related rehospitalization during the follow‐up period. If medical records were inconclusive or not available, a telephone interview with the next of kin was used to ascertain or confirm the circumstances leading to death. An end‐point committee consisting of 2 cardiologists (M.H.S. and C.O.) blinded to HADS anxiety and depression scores reviewed medical records and telephone interviews to determine cause of death. A consensus was reached for each case. All 934 patients were included in the mortality analyses, with 22 lost during the follow‐up period. Among these 22, 2 were lost within the first month, and the other 20 withdrew consent or were lost to follow‐up after a mean of 1.2±0.7 years from the time of their baseline assessment. The 22 patients with missing follow‐up data were comparable to the others in all other demographics and medical history variables, including scores on the HADS anxiety scale (complete follow‐up, 4.4±4.0; partial follow‐up, 4.8±4.8) and the HADS depression scale (complete follow‐up, 4.3±4.1; partial follow‐up, 3.3±4.1).

### Analytic Methods

Cox proportional hazards models were used to examine the association between baseline characteristics and mortality during a median follow‐up period of 3 years. The primary end point was time until death. Anxiety and depression were identified using the cutoff scores shown to have optimal sensitivity and specificity for detecting anxiety disorders and clinical depression (anxiety cases, HADS‐A scores ≥8; depression cases, HADS ‐D scores ≥8).^32^ In the originally planned model, age, congestive heart failure (CHF), presence of 3‐vessel coronary artery disease, LVEF, and renal disease were evaluated with anxiety and/or depression, both separately and together. None of the explanatory variables in the planned model had any missing data. In addition to evaluating the association with all‐cause mortality, analyses were repeated to evaluate the association between anxiety and depression and cardiovascular mortality, the most common cause of death. These analyses included all observations and treated the noncardiovascular deaths as censored observations.

To determine the robustness of the planned model, analyses considered other potentially relevant factors (sex, diabetes, internal cardioverter defibrillator [ICD], coronary artery bypass graft [CABG] treatment, smoking, diuretic use, insulin use, antidepressant use, benzodiazepine use, education status, and hypertension history), which were made available for possible entry into the model by stepwise selection (significance level required for entry into the model: *P*≤0.10). The only role of these extended analyses was to examine whether other factors modified the interpretation of anxiety and depression as explanatory because the analysis was not intended to develop a predictive model for patient outcome. For this expanded model, none of the variables had any missing data except for education, which was only missing 4 observations, and these missing variables had no influence on the findings. We repeated the analyses evaluating HADS‐A and HADS‐D scores as continuous variables, with scores on the HADS‐A or HADS‐D scale divided by 5 in order to produce more meaningful units of description for hazard ratios. In addition, we evaluated risk across quartiles of HADS‐A and HADS‐D scores to examine whether each was related to mortality risk in a graded fashion. We also evaluated how anxiety and depression work together to predict mortality risk by fitting models that included their interaction. These models not only enabled evaluation of the interaction but also provided hazard ratios for anxiety only contrasted both with patients with neither anxiety nor depression and patients with depression only. Similarly, these models provided hazard ratios for depression only contrasted both with patients with neither anxiety nor depression and patients with anxiety only. These models also enabled the evaluation of the hazard ratio for comorbid anxiety and depression versus the absence of both.

## Results

The patient sample was 70% male and 79% white, with a mean age of 62 years (range, 29 to 90 years; see Table 1). Scores ranged from 0 to 19 on the HADS‐A subscale and from 0 to 21 on the HADS‐D subscale. Approximately 20% of the sample reported HADS‐A or HADS‐D scores at or above the cutoff of 8 recommended for identifying clinically significant anxiety and depression. HADS‐A and HADS‐D scores were significantly correlated (*r*=0.66, *P*<0.001), and 60% of the cases classified as depressed also met criteria for elevated anxiety, and 52% of the anxiety cohort met criteria for elevated depression. When evaluated in the full cohort, both HADS‐A and HADS‐D scores were associated with younger age (anxiety: *r*=−0.25, *P*<0.001; depression: *r*=−0.18, *P*<0.001) and higher rates of smoking (anxiety: *r*=0.13, *P*<0.001; depression: *r*=0.15, *P*<0.001). However, only the HADS anxiety scores were associated with reduced sleep duration (*r*=−0.09, *P*=0.016), female sex (*r*=0.07, *P*=0.032), greater body mass index (*r*=0.07, *P*=0.027), and hypertension history (*r*=0.06, *P*=0.056). Similarly, only the HADS depression scores were associated with congestive heart failure (*r*=0.13, *P*<0.001) and lower LVEF (*r*=−0.07, *P*=0.037). When groups with anxiety only and depression only were contrasted with patients without elevated symptoms of anxiety or depression (see Table 1), anxiety's association with reduced sleep duration and female sex were retained, but anxiety was no longer associated with smoking and was associated with lower alcohol consumption (see Table 1).

**Table 1.** Demographic and Clinical Characteristics of Study Groups

	Neither (n=680)	Anxiety* Only (n=90)	Depression* Only (n=65)	Anxiety* and Depression* (n=99)
Demographics				
Female, %	29	43*	26	32
Age, y	63±11	60±10	62±11	55±11*
Minority, %	21	18	11	29
Obese, %	39	48	49	43
BMI, kg/m^2^	29.5±5.9	31.3±7.8*	31.5±9.0*	29.6±7.3
<HS education, %	31	36	31	34
Daily sleep, h	7.2±2.0	6.6±2.1*	7.1±3.5	6.8±2.7
Alcohol consumption,%				
Never	58	74*	62	65
Low*	24	18	23	25
Moderate‐High*	18	8*	15	10
Risk factors and medical history				
Current smokers, %	12	11	22	32*
HTN history, %	78	86	85	80
SBP	130±17	131±17	128±17	125±18*
DBP	69±10	70±11	69±12	67±11
Diabetes history, %	36	43	37	42
MI history, %	42	37	49	51
3‐Vessel CAD, %	46	43	37	30*
CABG treatment, %	29	31	25	28
LVEF≤40%, %	16	18	26	26*
CHF, %	18	24	35*	29*
ICD, %	2	7*	6	6
PVD, %	24	27	20	27
CVD, %	16	17	25	15
COPD, %	15	19	23	17
PUD, %	11	10	5	8
Renal disease, %	15	10	11	13
Prognostic medications, %				
Benzodiazepines	9	18*	18	19*
Antidepressants	18	21	34*	30*
Insulin	15	21	18	25*
Diuretics	34	41	52*	37
Psychosocial assessments				
HADS anxiety score	3±2	10±2*	4±2*	12±3*
HADS depression score	2±2	5±2*	10±2*	13±4*
All‐cause mortality,%	11.5	17	18.5	29*
High rehospitalization rate*, %	17	26	17	33*

Contrasts are with patients with HADS‐A and HADS‐D scores <8; sleep duration available only in a subset of 791 patients; alcohol use available only in a subset of 807 patients. BMI indicates body mass index; HS, high school; HTN, hypertension; SBP, systolic blood pressure; DBP, diastolic blood pressure; MI, myocardial infarction; CAD, coronary artery disease; CABG, coronary artery bypass graft; LVEF, left ventricular ejection fraction; CHF, congestive heart failure; ICD, implantable cardioverter defibrillator; PVD, peripheral vascular disease; CVD, cardiovascular disease; COPD, chronic obstructive pulmonary disease; PUD, peptic ulcer disease; HADS, Hospital Anxiety and Depression Scale.

* *P*≤0.05.

* *P*≤0.01.

* *P*≤0.001.

* HADS‐A scores ≥8.

* HADS‐D scores ≥8.

* >1 Rehospitalization per year during follow‐up.

* <2 Drinks per month.

* Daily alcohol consumption.

### Association of Anxiety and Depression With All‐Cause Mortality

Over the 3‐year follow‐up period, there were 133 deaths. Twenty‐two deaths were adjudicated to be related to a medical procedure occurring during hospitalization at baseline or hospitalization during the follow‐up period. Of these 22 deaths, 5 met criteria for elevated anxiety, and 7 met criteria for elevated depression.

Elevated anxiety or depression symptoms, defined using the predetermined cutoff points of 8, recommended by Zigmond and Snaith to identify clinical cases of anxiety and depression,^32^ were separately associated with mortality after adjusting for variables in the original planned model, which included age, CHF, 3‐vessel coronary artery disease, LVEF, and renal disease (elevated anxiety HR, 2.27; 95% CI, 1.55 to 3.33; *P*<0.001; elevated depression HR, 2.18; 95% CI, 1.47 to 3.22; *P*<0.001). Although dichotomization at 8 has been found to have the highest sensitivity and specificity to identify anxiety disorders and depression in primary care patients and noncancer medical patients, a cutoff of 9 on the HADS‐A subscale has been found to be optimal to identify anxiety disorders in some patient populations such as cancer patients (whereas a cutoff point of 8 on the HADS‐D subscale remained optimal for depression in this population).^34^ When HADS‐A scores were dichotomized at 9, anxiety continued to be associated with mortality (HR, 2.63; 95% CI, 1.73 to 3.99; *P*<0.001).

The opportunity provided by stepwise selection of all other baseline characteristics extended the planned model by also including ICD, antidepressant use, and insulin use. No other variables were selected for the model through stepwise selection. In this extended model, anxiety scores of ≥8 continued to be associated with a 2‐fold increased risk of mortality (HR, 2.00; 95% CI, 1.35 to 2.97; *P*<0.001), and similar findings were observed with depression (HR, 1.69; 95% CI, 1.13 to 2.54; *P*=0.011). These results support the robustness of the findings for anxiety and depression from the planned model.

### Anxiety and All‐Cause Mortality in the Context of Comorbid Depression

Including both depression and anxiety status in the planned model did not eliminate the association between anxiety and mortality (HR, 1.83; 95% CI, 1.18 to 2.83 *P*=0.006) or the association between depression and mortality (HR, 1.66; 95% CI, 1.06 to 2.58; *P*=0.025; see Table 2). However, in the model extended by stepwise selection and containing both depression and anxiety, anxiety continued to be associated with mortality (HR, 1.79; 95% CI, 1.15 to 2.78; *P*=0.010), but the association of depression with mortality was attenuated (HR, 1.31; 95% CI, 0.82 to 2.05; *P*=0.27).

**Table 2.** Cox Proportional Regression Analyses for All‐Cause Mortality

Variable	Planned Model* HR (95% CI)	*P* Value	Extended Model* HR (95% CI)	*P* Value
Anxiety	1.83 (1.18 to 2.83)	0.006	1.79 (1.15 to 2.78)	0.010
Depression	1.66 (1.06 to 2.58)	0.025	1.31 (0.82 to 2.05)	0.27
Age tertile	1.66 (1.32 to 2.09)	<0.001	1.83 (1.45 to 2.32)	<0.001
CHF	2.44 (1.62 to 3.68)	<0.001	2.15 (1.42 to 3.28)	<0.001
3‐Vessel CAD	1.78 (1.24 to 2.54)	0.002	1.85 (1.29 to 2.65)	<0.001
LVEF	0.99 (0.97 to 1.00)	0.063	0.99 (0.97 to 1.00)	0.065
Renal disease	2.22 (1.51 to 3.27)	<0.001	2.19 (1.46 to 3.28)	<0.001
ICD	—		5.49 (3.23 to 9.33)	<0.001
Antidepressant use	—		1.90 (1.29 to 2.80)	0.001
Insulin			1.59 (1.06 to 2.38)	0.025

HR indicates hazard ratio; CI, confidence interval; CHF, congestive heart failure; CAD, coronary artery disease; LVEF, left ventricular ejection fraction; ICD, implantable cardioverter defibrillator.

* Adjusted for age tertile, CHF, presence of 3‐vessel disease, left ventricular ejection fraction, and renal disease (end‐stage renal disease and chronic renal insufficiency).

* Adjusted for the variables in the a priori planned model, as well as presence of an internal cardioverter defibrillator, antidepressant use, and insulin use.

For both the planned and the extended models, evaluation of an interaction term for anxiety and depression showed that it was not explanatory (planned model, *P*=0.70; extended model, *P*=0.66). Nevertheless, inclusion of the interaction term for anxiety and depression is of interest for showing the similarity of the association between anxiety and mortality when contrasted with patients with neither anxiety nor depression (planned model HR, 1.71; 95% CI, 0.98 to 3.00; *P*=0.060) and when contrasted with patients with depression only (planned model HR, 2.04; 95% CI, 1.00 to 4.17; *P*=0.049; see Table 3). Similarly, inclusion of the interaction term showed the similarity of the association between depression and mortality when contrasted with patients with neither anxiety nor depression (planned model HR, 1.52; 95% CI, 0.80 to 2.88; *P*=0.20) and when contrasted with patients with anxiety only (planned model HR, 1.81; 95% CI, 0.96 to 3.43; *P*=0.070). Moreover, this model showed a 3‐fold increased risk of mortality for patients with comorbid anxiety and depression versus those with neither (planned HR, 3.10; 95% CI, 1.95 to 4.94; *P*<0.001), with this increased risk being comparable to that produced by the product of the separate hazard ratios for anxiety and depression in the planned model without the interaction term for anxiety and depression (HR=1.83×1.66=3.04; 95% CI, 1.93 to 4.79; *P*<0.001).

**Table 3.** Hazard Ratios and 95% Confidence Intervals for All‐Cause Mortality

	Neither Anxiety nor Depression (n=680)	Anxiety Only (n=90)	Depression Only (n=65)	Anxiety and Depression (n=99)
Number of deaths	78	15	12	28
Planned Model HR*				
Versus neither	1.00 (ref)	1.71 (0.98 to 3.00)	1.52 (0.80 to 2.88)	3.10 (1.95 to 4.94)
Versus anxiety alone	NA	1.00 (ref)	NA	1.81 (0.96 to 3.43)
Versus depression alone	NA	NA	1.00 (ref)	2.04 (1.00 to 4.17)

HR indicates hazard ratio; CI, confidence interval; ref, reference; CHF, congestive heart failure.

* Adjusted for age tertile, CHF, presence of 3‐vessel disease, left ventricular ejection fraction, and renal disease (end‐stage renal disease and chronic renal insufficiency).

### Graded Association of Anxiety and Depression

**Figure 2.**
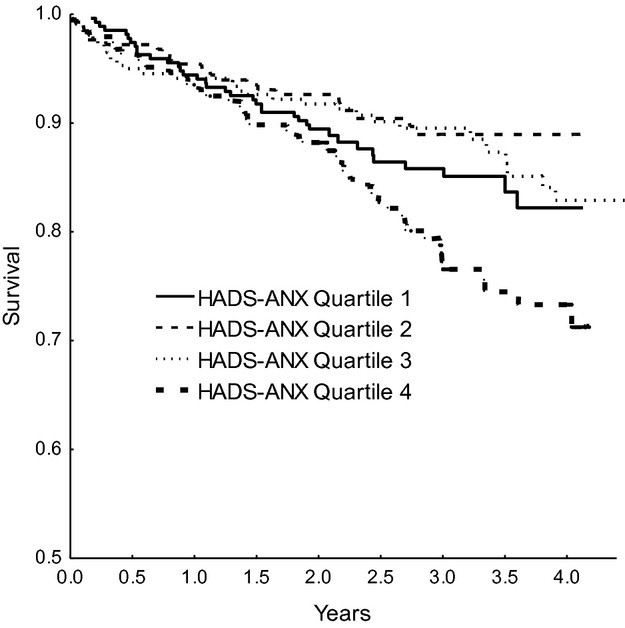
Hazard plot of the association between quartile of Hospital Anxiety and Depression Scale–Anxiety (HADS‐A) score and mortality.

**Figure 3.**
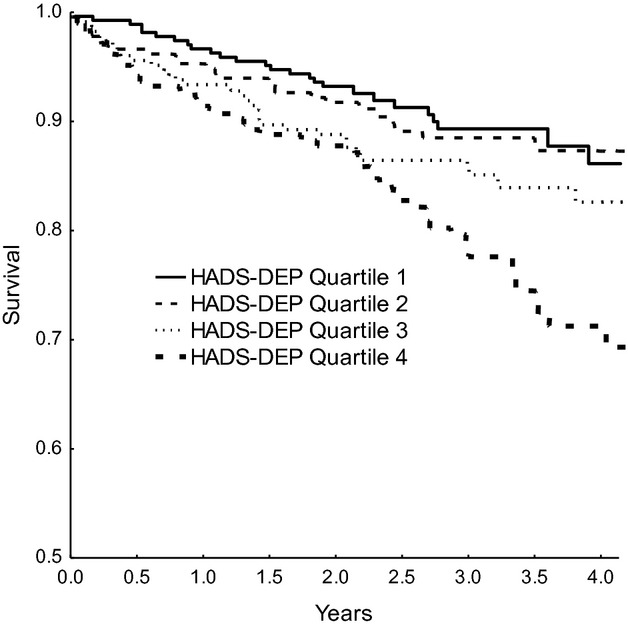
Hazard plot of the association between quartile of Hospital Anxiety and Depression Scale–Depression (HADS‐D) score and mortality.

An association with all‐cause mortality was also observed when either anxiety or depression was represented in the planned model using continuous scores on the HADS anxiety subscale (HADS anxiety score/5 HR, 1.59; 95% CI, 1.30 to 1.96; *P*<0.001) or continuous scores on the HADS depression subscale (HADS depression score/5 HR, 1.54; 95% CI, 1.29 to 1.85; *P*<0.001). Including both anxiety and depression scores/5 in the same model resulted in some attenuation of the association between anxiety and mortality (HR, 1.29; 95% CI, 0.98 to 1.70; *P*=0.069) but not between depression and mortality (HR, 1.33; 95% CI, 1.04 to 1.70; *P*=0.024). Evaluation of an interaction term for anxiety and depression showed that it was not explanatory (*P*=0.97). As shown descriptively using survival curves, an association with all‐cause mortality was observed for quartiles of anxiety and depression in the setting of no adjustment for baseline characteristics (see Figures 2 and 3). A graded increase in mortality was observed with increasing quartile of HADS‐D score. In contrast, the association between anxiety and risk appeared to be driven largely by the fourth quartile of scores, which corresponds to participants with anxiety values of ≥8.

### Association of Anxiety and Depression With Cardiovascular Mortality

The majority of deaths in the current cohort were attributed to cardiovascular causes (93 of the 133). Elevated anxiety and depression (HADS scores ≥8) showed similar associations with cardiovascular deaths as observed with all‐cause mortality when evaluated separately (anxiety HR, 2.11; 95% CI, 1.33 to 3.35; *P*=0.002; depression HR, 2.02; 95% CI, 1.26 to 3.24; *P*=0.004) and when included in the same model together (anxiety HR, 1.75; 95% CI, 1.04 to 2.94; *P*=0.036; depression HR, 1.58; 95% CI, 0.93 to 2.69; *P*=0.093). There were no significant interactions between anxiety and depression in these models.

## Discussion

Anxiety is common in Western societies, with approximately 30% of US adults reporting a clinically significant anxiety disorder during their lifetimes.^42^ In the current cohort of CHD patients, those with elevated anxiety symptoms showed a 2‐fold increased risk of mortality, suggesting that anxiety has an explanatory role that may be clinically important in the identification of patients at higher risk of mortality. The increased mortality risk was most pronounced in the patient group with comorbid anxiety and depression, in whom the risk of mortality was 3‐fold higher than in patients with neither anxiety nor depression.

### Association of Anxiety With All‐Cause Mortality

The current findings that elevated anxiety symptoms were associated with a 2‐fold increased risk of mortality in CHD patients are consistent with previous reports of an approximate 2‐fold increased risk of mortality associated with anxiety in hospitalized patients undergoing CABG^21,24^ and in cardiac patients tested outside the hospital environment.^20,23,25^ For example, Frasure‐Smith and colleagues^23^ reported that CHD patients with generalized anxiety disorder (GAD) assessed 2 months following hospital discharge for acute coronary syndrome showed a 2.3‐fold increased risk of adverse cardiac events, and Strik et al^25^ reported a 2.8‐fold increased risk of adverse events in acute post‐MI patients in whom anxiety was measured 1 month following hospital discharge. Similarly, a 2‐fold increased risk of adverse events was also found in cardiac patients with stable coronary disease undergoing rehabilitation^20^ and in patients with consistently elevated anxiety during annual clinic visits.^26^ However, anxiety has not been found to be related to mortality risk in cardiac patients evaluated during hospitalization for an acute myocardial infarction.^28–31^ In addition, in a sample of 4864 patients undergoing exercise stress testing, elevated anxiety was predictive of increased risk of mortality only in those patients with left ventricular dysfunction. In the remaining patients, anxiety assessed at the time of stress testing was found to be associated with improved survival.^43^ These findings suggest that anxiety may convey some protective effects, possibility through increased motivation to seek and comply with health‐promoting behavior, despite its association with overall health‐damaging consequences in patient populations.

Methodological differences in anxiety assessment may have contributed to the apparent discrepant findings between studies. For example, in hospitalized patients following a major event such as MI, estimation of steady‐state levels of anxiety was made difficult by the substantial fluctuation of anxiety during hospitalization following MI.^44–45^ Furthermore, anxiety is often measured using self‐report questionnaires that are not designed to be used in elderly hospitalized patients. In addition to failing to differentiate anxiety from symptoms related to physical disorders, such as dizziness, insomnia, and fatigue, some of the commonly used questionnaires may be inferior to other anxiety measures in terms of discriminant and factorial validity^46–47^ and may be especially problematic when used to differentiate elderly patients with diagnosed anxiety disorder from nonanxious patients.^47–48^

### Anxiety and Mortality in the Context of Comorbid Depression

The present findings that more than half the patients with elevated HADS‐A scores consistent with clinically significant anxiety (HADS‐A≥8) also met criteria for clinical depression defined as HADS‐D score ≥8 are consistent with similar estimates of high comorbidity between GAD and unipolar depression.^49–50^ For example, 58% of the respondents in the National Comorbidity Survey and 70% of respondents in the Midlife Development in the United States Survey with generalized anxiety disorder in the previous 12 months also met criteria for major depression. Similarly, approximately two‐thirds of patients with panic disorder or agoraphobia fulfilled criteria for lifetime major depression.^51^

Patients with comorbid anxiety and depression have been reported to show a more chronic course of psychopathology, with significantly greater functional impairment and more resistance to psychiatric treatment than patients with either condition alone.^49,52–54^ Findings from recent twin studies suggest that a genetic predisposition toward a negative appraisal of life events may be present in both anxiety and depression. The presence of this genetic predisposition may lead to amplification of the perceived stressfulness of major life events. The present findings of an additive association between anxiety and depression in relation to mortality risk are consistent with greater chronicity of anxiety and depression and suggest that future research should evaluate the efficacy of stress management or cognitive behavior therapy in this patient population.

Despite the extensive comorbidity between anxiety and depression, few studies have accounted for the impact of the alternate psychosocial factor when evaluating the prognostic significance of anxiety or depression in CHD patients. The findings of an approximately 3‐fold increased risk of mortality in patients with comorbid anxiety and depression are consistent with previous findings of a combined action between anxiety and depression in the Vietnam Experience Study, in which veterans with comorbid major depressive disorder (MDD) and GAD were at substantially greater risk of mortality than the veterans who reported only MDD or GAD.^55^ Similar evidence for a combined action between anxiety and depression was reported in the Olmstead County study, in which the presence of anxiety and depression conferred greater risk of rehospitalization than either factor alone^56^and in the PROMOTION study, a randomized clinical trial designed to evaluate the efficacy of an education intervention in decreasing patient delay in seeking treatment for CHD symptoms. Patients enrolled in PROMOTION with depression or anxiety alone were not at greater risk of mortality; however, those with persistent comorbid symptoms had significantly higher mortality.^57^ In addition, 1 previous study evaluating the impact of anxiety and depression reported that combining the number of psychosocial risk factors (ie, anxiety, depression, or depression history) resulted in a cumulative relationship for a composite‐outcome end point defined by rehospitalization, MI, and cardiac mortality in 222 acute post‐MI patients.^22^ In contrast, a combined effect of anxiety and depression was not found in a study of 804 CHD patients, in which the association between generalized anxiety disorder (GAD) and clinical depression on a composite index of risk was evaluated.^23^ Although reduced power may explain the lack of a combined effect of anxiety and depression in this study, because only 11 patients were diagnosed with both GAD and depression in this study, there was also no apparent combined effect when anxiety and depressive symptoms were measured using the HADS anxiety scale to assess anxiety and the Beck Depression Inventory to measure depression.

### Study Limitations

Caution must be taken in generalizing the current findings to all CHD patients for a number of reasons. First, patients who undergo diagnostic tests such as cardiac catheterization to explain physical symptoms may be more likely to include individuals with elevated anxiety and depression.^58^ Second, the circumstances associated with the hospitalization or catheterization procedure may have influenced the assessment of anxiety or depression in some individuals. In addition, although the HADS was developed specifically to classify cases of anxiety and depression in a medical setting, it may offer some disadvantages to the use of a diagnostic interview to define clinically significant anxiety and depression. Evaluation of the association between psychosocial factors and risk of mortality may also have been compromised in this sample by the higher number of deaths occurring as a direct consequence of a medical procedure. Exclusion of the procedural deaths, however, did not alter the strength of the association between elevated anxiety or elevated depression and mortality risk when these factors were evaluated separately. When evaluated together, the association between anxiety and mortality was maintained (HR, 2.07; 95% CI, 1.30 to 3.30; *P*=0.002), but depression had an attenuated association with increased risk of mortality when the analysis was limited to nonprocedural deaths (HR, 1.50; 95% CI, 0.93 to 2.44; *P*=0.10). The findings were also limited in that substance abuse was not considered a potential confounding variable. A number of studies have found high rates of substance use and abuse in clinically depressed patients and in some types of anxiety disorders such as posttraumatic stress disorder.^59–61^ However, we did examine smoking and alcohol use in the current cohort and observed an association between current smoking and depressive symptoms. In contrast, anxiety was unrelated to smoking and was related to significantly lower alcohol use. It therefore seems plausible that substance abuse would not explain the association between anxiety and mortality in the current cohort but could have contributed to the excess mortality observed in the patients with depression.

## Conclusions

Anxiety and depression are common in CHD patients during hospitalization for coronary angiography, and each was independently associated with an approximate 2‐fold increased risk of all‐cause mortality. The association of these factors was additive, with a 3‐fold increased risk in anxious patients with comorbid depression. These findings suggest that clinicians assess patients for both anxiety and depression and continue to monitor these symptoms on a regular basis. Patients with elevated anxiety, particularly when found in the context of comorbid depression, may benefit from treatment of anxiety and from more intensive monitoring.

## References

[1] van Melle LP De Jonge P Spijkerman TA Tijssen JGP Ormel J van Veldhuisen DJ van den Brink RH van de Berg MP Prognostic association of depression following myocardial infarction with mortality and cardiovascular events: a meta‐analysis. Psychosom Med. 2004; 66:814-822 15564344 10.1097/01.psy.0000146294.82810.9c

[2] Barefoot JC Helms MJ Mark DB Blumenthal JA Califf RM Haney TL O'Connor CM Siegler IC Williams RB Depression and long‐term mortality risk in patients with coronary artery disease. Am J Cardiol. 1996; 78:613-617 8831391 10.1016/s0002-9149(96)00380-3

[3] Frasure‐Smith N Lesperance F Talajic M Depression and 18‐month prognosis after myocardial infarction. Circulation. 1995; 91:999-1005 7531624 10.1161/01.cir.91.4.999

[4] Breir A Charney DS Heninger GR Major depression in patients with agoraphobia and panic disorder. Arch Gen Psychiatry. 1984; 41:1129-1135 6508503 10.1001/archpsyc.1984.01790230015002

[5] Kessler RC Chiu WT Demler O Walters EE Prevalence, severity, and comorbidity of twelve‐month DSM‐IV disorders in the National Comorbidity Survey Replication (NCS‐R). Arch Gen Psychiatry. 2005; 62:617-627 15939839 10.1001/archpsyc.62.6.617 PMC2847357

[6] Kessler RC Nelson CB McGonagle KA Liu J Swartz M Blazer DG Comorbidity of DSM‐III‐R major depressive disorder in the general population: results from the US National Comorbidity Survey. Br J Psychiatry Suppl. 1996; 168:17-30 8864145

[7] Hoehn‐Saric R McLeod DR The peripheral sympathetic nervous system: its role in pathological anxiety. Psychiatr Clin North Am. 1988; 11:375-386 3047706

[8] Nesse RM Cameron OG Curtis GC McCann DS Huber‐Smith MJ Adrenergic function in patients with panic anxiety. Arch Gen Psychiatry. 1984; 41:771-776 6331337 10.1001/archpsyc.1984.01790190045005

[9] Alvarenga ME Richards JC Lambert G Esler MD Psychophysiological mechanisms in panic disorder. A correlative analysis of noradrenaline spillover, power spectral analysis of heart rate variability and psychological variables. Psychosom Med. 2006; 68:8-16 16449406 10.1097/01.psy.0000195872.00987.db

[10] Pitsavos C Panagiotakos DB Papageorgiou C Tsetsekou E Soldatos C Stefanadis C Anxiety in relation to inflammation and coagulation markers, among healthy adults: the ATTICA Study. Atherosclerosis. 2006; 185:320-326 16005881 10.1016/j.atherosclerosis.2005.06.001

[11] Bankier B Barajas J Martinez‐Rumayor A Januzzi JL Association between C‐reactive protein and generalized anxiety disorder in stable coronary heart disease patients. Eur Heart J. 2008; 29:2212-2217 18603621 10.1093/eurheartj/ehn326

[12] O'Donovan A Hughes BM Slavich GM Lynch L Cronin M O'Farrelly C Malone KM Clinical anxiety, cortisol and interleukin‐6: evidence for specificity in emotion‐biology relationships. Brain Behav Immun. 2010; 24:1074-1077 20227485 10.1016/j.bbi.2010.03.003 PMC4361085

[13] Brennan AM Fargnoli JL Williams CJ Li T Willett W Kawachi I Qi L Hu FB Mantzoros CS Phobic anxiety is associated with higher serum concentrations of adipokines and cytokines in women with diabetes. Diabetes Care. 2009; 32:926-931 19223611 10.2337/dc08-1979 PMC2671103

[14] Markovitz JH Matthews KA Kannel WB Cobb JL D'Agostino RB Psychological predictors of hypertension in the Framingham Study. JAMA. 1993; 270:2439-2443 8230620

[15] Smoller JW Pollack MH Wassertheil‐Smoller S Jackson RD Oberman A Wong ND Sheps D Panic attacks and risk of incident cardiovascular events among postmenopausal women in the women's health initiative observational study. Arch Gen Psychiatry. 2007; 64:1153-1160 17909127 10.1001/archpsyc.64.10.1153

[16] Albert CM Chae CU Rexrode KM Manson JE Kawachi I Phobic anxiety and risk of coronary heart disease and sudden cardiac death among women. Circulation. 2005; 111:480-487 15687137 10.1161/01.CIR.0000153813.64165.5D

[17] Haines AP Imeson JD Meade TW Phobic anxiety and ischaemic heart disease. Br Med J. 1987; 295:297-299 3115417 10.1136/bmj.295.6593.297 PMC1247140

[18] Kawachi I Colditz GA Ascherio A Rimm EB Giovannucci E Stampfer MJ Willett WC Prospective study of phobic anxiety and risk of coronary heart disease in men. Circulation. 1994; 89:1992-1997 8181122 10.1161/01.cir.89.5.1992

[19] Kawachi I Sparrow D Vokonas PS Weiss ST Symptoms of anxiety and risk of coronary heart disease: the normative aging study. Circulation. 1994; 90:2225-2229 7955177 10.1161/01.cir.90.5.2225

[20] Rothenbacher D Hahmann H Wusten B Koenig W Brenner H Symptoms of anxiety and depression in patients with stable coronary heart disease: prognostic value and consideration of pathogenetic links. Eur J Cardiovasc Prev Rehabil. 2007; 14:547-554 17667646 10.1097/HJR.0b013e3280142a02

[21] Tully PJ Baker RA Knight JL Anxiety and depression as risk factors for mortality after coronary artery bypass surgery. J Psychosom Res. 2008; 64:285-290 18291243 10.1016/j.jpsychores.2007.09.007

[22] Frasure‐Smith N Lesperance F Talajic M The impact of negative emotions on prognosis following myocardial infarction: is it more than depression? Health Psychol. 1995; 14:388-398 7498109 10.1037//0278-6133.14.5.388

[23] Frasure‐Smith N Lesperance F Depression and anxiety as predictors of 2‐year cardiac events in patients with stable coronary artery disease. Arch Gen Psychiatry. 2008; 65:62-71 18180430 10.1001/archgenpsychiatry.2007.4

[24] Szekely A Balog P Benko E Breuer T Szekely J Kertai MD Horkay F Kopp MS Thayer JF Anxiety predicts mortality and morbidity after coronary artery and valve surgery–a 4 year follow‐up study. Psychosom Med. 2007; 69:625-631 17724254 10.1097/PSY.0b013e31814b8c0f

[25] Strik JJ Denollet J Lousberg R Honig A Comparing symptoms of depression and anxiety as predictors of cardiac events and increased health care consumption after myocardial infarction. J Am Coll Cardiol. 2003; 42:1801-1807 14642691 10.1016/j.jacc.2003.07.007

[26] Shibeshi WA Young‐Xu Y Blatt CM Anxiety worsens prognosis in patients with coronary artery disease. J Am Coll Cardiol. 2007; 49:2021-2027 17512358 10.1016/j.jacc.2007.03.007

[27] Roest AM Martens EJ Denollet J De Jonge P Prognostic association of anxiety post myocardial infarction with mortality and new cardiac events: a meta‐analysis. Psychosom Med. 2010; 72:563-569 20410247 10.1097/PSY.0b013e3181dbff97

[28] Ahern DK Gorkin L Anderson JL Tierney C Hallstrom A Ewart C Capone RJ Schron E Kornfeld D Herd JA Richardson DW Follick MJ Biobehavioral variables and mortality or cardiac arrest in the cardiac arrhythmia pilot study (CAPS). Am J Cardiol. 1990; 66:59-62 2193497 10.1016/0002-9149(90)90736-k

[29] Frasure‐Smith N Lesperance F Depression and other psychological risks following myocardial infarction. Arch Gen Psychiatry. 2003; 60:627-636 12796226 10.1001/archpsyc.60.6.627

[30] Kornerup H Zwisler AO Prescott E The CANREHAB Group No association between anxiety and depression and adverse clinical outcome among patients with cardiovascular disease: findings from the DANREHAB trial. J Psychosom Res. 2011; 71:207-214 21911097 10.1016/j.jpsychores.2011.04.006

[31] Lane D Carroll D Ring C Beevers DG Lip GYH Mortality and quality‐of‐life twelve months after myocardial infarction: effects of depression and anxiety. Psychosom Med. 2001; 63:221-230 11292269 10.1097/00006842-200103000-00005

[32] Zigmond AS Snaith RP The hospital anxiety and depression scale. Acta Psychiatr Scand. 1983; 67:361-370 6880820 10.1111/j.1600-0447.1983.tb09716.x

[33] Blair SN Haskell WL Ho P Paffenbarger RS Vranizan KM Farquhar JW Wood PD Assessment of habitual physical activity by a seven‐day recall in a community survey and controlled experiments. Am J Epidemiol. 1985; 122:794-804 3876763 10.1093/oxfordjournals.aje.a114163

[34] Bjelland I Dahl AA Haug TT Neckelmann D The validity of the Hospital Anxiety and Depression Scale: an updated literature review. J Psychosom Res. 2002; 52:69-77 11832252 10.1016/s0022-3999(01)00296-3

[35] Moorey S Greer S Watson M Gorman C Rowden L Tunmore R Robertson B Bliss J The factor structure and factor stability of the hospital anxiety and depression scale in patients with cancer. Br J Psychiatry. 1991; 158:255-259 1812841 10.1192/bjp.158.2.255

[36] Dunbar M Ford G Hunt K Der G A confirmatory factor analysis of the Hospital Anxiety and Depression Scale: comparing empirically and theoretically derived structures. Br J Clin Psychol. 2000; 39:79-94 10789030 10.1348/014466500163121

[37] Dagnan D Chadwick P Trower P Psychometric properties of the Hospital Anxiety and Depression Scale with a population of members of a depression self‐help group. Br J Med Psychol. 2000; 73:129-137 10759056 10.1348/000711200160255

[38] Hinz A Braehler E Normative values for the Hospital Anxiety and Depression Scale (HADS) in the general German population. J Psychosom Res. 2011; 71:74-78 21767686 10.1016/j.jpsychores.2011.01.005

[39] Hicks JA Jenkins JG The measurement of preoperative anxiety. J R Soc Med. 1988; 81:517-519 3184108 10.1177/014107688808100907 PMC1291760

[40] Wilkinson MJ Barczak P Psychiatric screening in general practice: comparison of the general health questionnaire and the hospital anxiety depression scale. J R Coll Gen Pract. 1988; 38:311-313 3255827 PMC1711493

[41] Hopwood P Howell A Maguire P Screening for psychiatric morbidity in patients with advanced breat cancer: validation of two self‐report questionnaires. Br J Cancer. 1991; 646:353-356 1892763 10.1038/bjc.1991.305 PMC1977509

[42] Kessler RC Berglund P Demler O Jin R Merikangas KR Walters EE Lifetime prevalence and age‐of‐onset distributions of DSM‐IV disorders in the National Comorbidity Survey Replication. Arch Gen Psychiatry. 2005; 62:593-602 15939837 10.1001/archpsyc.62.6.593

[43] Meyer T Buss U Herrmann‐Lingen C Role of cardiac disease severity in the predictive value of anxiety for all‐cause mortality. Psychosom Med. 2010; 72:9-15 19995887 10.1097/PSY.0b013e3181c64fc0

[44] Philip AE Cay EL Vetter NJ Short‐term fluctuations in anxiety in patients with myocardial infarction. J Psychosom Res. 1979; 23:277-280 529201 10.1016/0022-3999(79)90031-x

[45] Thompson DR Webster RA Cordle CJ Specific sources and patterns of anxiety in patients with myocardial infarction. Br J Med Psychol. 1987; 60:343-348 3426972 10.1111/j.2044-8341.1987.tb02753.x

[46] Kennedy BL Schwab JJ Morris RL Beldia G Assessment of state and trait anxiety in subjects with anxiety and depressive disorders. Psychiatr Q. 2001; 72:263-276 11467160 10.1023/a:1010305200087

[47] Kabacoff RI Segal DL Hersen M VanHasselt VB Psychometric properties and diagnostic utility of the Beck Anxiety Inventory and the State‐Trait Anxiety Inventory with older adult psychiatric inpatients. J Anxiety Disord. 1997; 11:33-47 9131880 10.1016/s0887-6185(96)00033-3

[48] Emoto M Nishizawa Y Kawagishi T Maekawa K Hiura Y Kanda H Izumotani K Shoji T Ishimura E Inaba M Okuno Y Morii H Stiffness indexes Β of the common carotid and femoral arteries are associated with insulin resistance in NIDDM. Diabetes Care. 1998; 21:1178-1183 9653616 10.2337/diacare.21.7.1178

[49] Judd LL Kessler RC Paulus MP Zeller PV Wittchen HU Kunovac JL Comorbidity as a fundamental feature of generalized anxiety disorders: results from the National Comorbidity Study (NCS). Acta Psychiatr Scand. 1998; 98suppl 393:6-11 10.1111/j.1600-0447.1998.tb05960.x 9777041

[50] Kessler RC Berglund PA Dewit DJ Ustun TB Wang PS Wittchen H Distinguishing generalized anxiety disorder from major depression: prevalence and impairment from current pure and comorbid disorders in the US and Ontario. Int J Methods Psychiatr Res. 2002; 11:99-111 12459823 10.1002/mpr.128 PMC6878424

[51] Kessler RC Stang PE Wittchen H Ustun B Roy‐Byrne PP Walters EE Lifetime panic‐depression comorbidity in the National Comorbidity Survey. Arch Gen Psychiatry. 1998; 55:801-808 9736006 10.1001/archpsyc.55.9.801

[52] Roy‐Byrne PP Stang P Wittchen H Ustun B Walters EE Kessler RC Lifetime panic‐depression comorbidity in the National Comorbidity Survey: association with symptoms, impairment, course and help‐seeking. Br J Psychiatry. 2000; 176:229-235 10755069 10.1192/bjp.176.3.229

[53] Zajecka JM Ross JS Management of comorbid anxiety and depression. J Clin Psychiatry. 1995; 56suppl 2:10-13 7844101

[54] Ghaemi SN Why antidepressants are not antidepressants: STEP‐BD, STAR*D, and the return of neurotic depression. Bipolar Disord. 2008; 10:957-968 19594510 10.1111/j.1399-5618.2008.00639.x

[55] Phillips AC Batty GD Gale CR Deary IJ Osborn D MacIntyre K Carroll D Generalized anxiety disorder, major depressive disorder, and their comorbidity as predictors of all‐cause and cardiovascular mortality: the Vietnam Experience Study. Psychosom Med. 2009; 71:395-403 19321850 10.1097/PSY.0b013e31819e6706

[56] Chamberlain AM Vickers KS Colligan RC Weston SA Rummans TA Roger VL Associations of preexisting depression and anxiety with hospitalization in patients with cardiovascular disease. Mayo Clin Proc. 2011; 86:1056-1062 22033250 10.4065/mcp.2011.0148 PMC3202995

[57] Doering LV Moser D Riegel B McKinley S Davidson P Baker H Meischke H Dracup K Persistent comorbid symptoms of depression and anxiety predict mortality in heart disease. Int J Cardiol. 2010; 145:188-192 19493579 10.1016/j.ijcard.2009.05.025 PMC2998562

[58] Katon W Lin EHB Kroenke K The association of depression and anxiety with medical symptom burden in patients with chronic medical illness. Gen Hosp Psychiatry. 2007; 29:147-155 17336664 10.1016/j.genhosppsych.2006.11.005

[59] Regier DA Farmer ME Rae DS Locke BZ Keith SJ Judd LL Goodwin FK Comorbidity of mental disorders with alcohol and other drug abuse; results from the Epidemiologic Catchment Area (ECA) Study. JAMA. 1990; 264:2511-2518 2232018

[60] Kessler RC Crum RM Warner LA Nelson CB Schulenberg J Anthony JC Lifetime co‐occurrence of DSM‐III‐R alcohol abuse and dependence with other psychiatric disorders in the National Comorbidity Survey. Arch Gen Psychiatry. 1997; 54:313-321 9107147 10.1001/archpsyc.1997.01830160031005

[61] Hofmann SG Richey JA Kashdan TB McKnight PE Anxiety disorders moderate the association between externalizing problems and substance use disorders: data from the National Comorbidity Survey—Revised. J Anxiety Disord. 2009; 23:529-534 19059752 10.1016/j.janxdis.2008.10.011

